# Subclinical Ketosis in Dairy Herds: Impact of Early Diagnosis and Treatment

**DOI:** 10.3389/fvets.2022.895468

**Published:** 2022-06-27

**Authors:** Giuseppe Cascone, Francesca Licitra, Alessandro Stamilla, Simona Amore, Mario Dipasquale, Rosario Salonia, Francesco Antoci, Alfonso Zecconi

**Affiliations:** ^1^Istituto Zooprofilattico Sperimentale of Sicily, Palermo, Italy; ^2^Department of Agricultural Food and Environmental Science (Di3A), University of Catania, Catania, Italy; ^3^R&D Laboratory, Leocata Mangimi, Modica, Italy; ^4^Department of Biomedical, Surgical and Dental Science, One Health Unit, University of Milan, Milan, Italy

**Keywords:** dairy cattle, milk quality, β-hydroxybutyrate (BHB), subclinical ketosis, propylene glycol

## Abstract

Clinical and subclinical ketosis (SCK) in dairy cows occurs during the lactation period frequently in many herds, causing a reduction in milk yield and alterations in milk quality with significant economic losses for farmers. SCK is defined as a preclinical stage of ketosis characterized by an elevated ketone body level without clinical signs. Often many cows develop an elevated ketone body level during the first weeks of lactation even though it never goes up to a critical point causing clinical signs. This study aimed to evaluate the prevalence of SCK in Sicily and assess the effect of a treatment with propylene glycol (PG) to control the SCK, thus, reducing the negative effect on milk quality yield. This cross-sectional study was carried out on 22 farms located south-east of Sicily and 1,588 cows in lactation. A total of 3,989 individual milk samples were collected from calving to 80 subsequently days to check the β-hydroxybutyrate (BHB) values in order to establish the SCK status by the Fourier Transform Infrared Spectroscopy. Moreover, the contents of fat, protein, lactose, casein, urea, somatic cell count and acetone were evaluated to identify a correlation between SCK and milk quality. A total of 1,100 cows showed BHB values higher than 0.10 mmol/L. These cows were considered SCK positive, were separated from the rest of the herd, and treated with PG (400 g/head per day), all SCK cows were treated with PG and cows without SCK were not treated. The results showed a prevalence of 41.5% of SCK-positive cows during the first 9 days of lactation. The comparison among the cure rate of treated cows shows that the treatment was most effective in the first 7 days of lactation (76.5% of treated cows) than in the following days. PG positively influenced the milk quality parameters, except for the fat proportion. Moreover, the animals treated with PG showed also an increase in milk yield, supporting the economical sustainability of treatment.

## Introduction

In the recent decades, the improvement in breeding of dairy cows such as the genetic selection and the new animal welfare standards positively impacted the milk production efficiency, leading also to a reduction in risk of an imbalance in the relationship between animal health and management. However, in the transition period, which runs from 3 weeks before to 3 weeks after calving, the dairy cows incur in one of the most critical phases of their productive life, because they can be subjected to various health problems (1, 2). Indeed, the significant and rapid increase in milk production with a relatively slow increase in dry matter intake (DMI), can lead to the establishment of a strong energy imbalance. The most common and evident consequence is lipomobilization with the release of non-esterified fatty acids (NEFAs) in the plasma. Under unfavorable metabolic conditions, starting from NEFA, the liver produces ketone bodies and in particular of β-hydroxybutyrate (BHB) then, when physiological limits are exceeded, it leads to ketosis or acetonemia (3). Different feeding strategies could represent a possible solution to modulate this dysmetabolism, seeking to promote an increase in dry matter intake and ensuring an adequate rumination time, because a reduction in rumination time, related to a less salivation, would induce a decrease in rumen pH with consequent rumen acidosis. Furthermore, a decrease in rumen pH could also affect the negative energy balance (NEB) (4). Moreover, the correct management of the cow groups, based on different nutrition programs, ensuring the free access to the resting and feeding areas and the temperature properly regulated (5), could also prevent clinical and subclinical ketosis. Mainly, ketosis clinical signs are related to a state of lethargy (lack of energy), a decrease in DMI, a reduction in milk production and quality and often a sweet smell on the breath, leading to significant economic losses for the farmers (6, 7). Subclinical ketosis (SCK) is defined as a preclinical stage of ketosis characterized by an elevate ketone body level without clinical signs. Often, SCK-positive milk samples are associated with an elevate value of somatic cell count (SCC) (8). In addition, during the preclinical stage of ketosis, the risk of contracting other metabolic disorders and even other pathologies, increases due to an impairment of the immune functions (9, 10). Therefore, the monitoring of postpartum SCK is essential to decrease the risk of clinical ketosis and related problems.

Although the importance of ketosis is wellknown, very few studies are focused on the prevalence of SCK in the Italian dairy herds (11), using the milk as diagnostic sample, and analyzing the related consequences in milk production and milk quality. The evaluation of the SCK prevalence in herd in early lactation period could help to set a treatment in order to raise the welfare standards and control the ketosis before the appearance of the first clinical signs (12–16). This study was designed as a cross-sectional study aimed to estimate SCK prevalence by the analysis of BHB values in milk samples as a robust method to SCK diagnosis, and to assess the SCK influence on fat, protein, lactose, casein, and somatic cells count (SCC) of milk, besides the milk yield. In addition, we were interested to assess the effects of a treatment with PG on the cure of SCK and on milk quality and yield by an uncontrolled longitudinal trial.

## Materials and Methods

This cross-sectional study was conducted in southeast of Sicily on 22 dairy cow herds for 15 months, from February 2019 to May 2020. A total of 3,989 milk samples from 1,588 dairy cows (primiparous and multiparous) were collected after calving, in the morning milking in order to establish the prevalence of SCK positive cows in herd and then assess the related risk factors. The number of samples collected, largely exceeds the minimum sample size calculated for a prevalence between 15 and 40% (17, 18) and a precision of 5%, which are, respectively, 200 and 400 samples. In total 13 of the 22 herds housed cows on deep litter, while the other nine housed cows on cubicles. In regard to nutrition, cows on 21 farms were fed with total mix ration (TMR) and cows on 1 farm with only hay and concentrate. In total, five different breeds were observed: Holstein Friesian 1,315 cows, (82.8%), Brown Swiss (157 cows, 9.9%), Jersey (6 cows, 0.4%), Simmental (80 cows, 5.0%) and crossbreed (30 cows, 1.9%). For further analysis, the last three categories were added and defined as mixed breeds (116 cows, 7.3%). The frequency of cows classified by parity was 28.6% (27.2–30.0%) first parity, 19.9% (18.6–21.1%) second parity, 14.9% (13.8–16.1%) third parity, 9.8% (8.9–10.7%) fourth parity, and 8.0% (7.2–8.9%) higher parities.

### Sampling

The samples were collected individually in a 50 ml tube from the whole milking of each animal, stored at 4°C and analyzed within 24 h by Milkoscan 7 and Fossomatic (FOSS, Hilleroed, Denmark). The sampling point was divided in 5 periods, in the first 21 days of lactation (DIM) the samples were collected once a week, after the 21st DIM was sampled only the milk of treated cows. The time periods are as following: A (1–9 DIM), B (10–15 DIM), C (16–21 DIM), D (22–30 DIM), and E (>30 DIM). The samples were classified based on breed, DIM, parity, and the daily milk production was recorded each sampling period (kg/head/day). Some cows were excluded from the study along the trial because they were treated with other antimicrobials or drugs related to other disorders, diseases, or dysmetabolism different than ketosis. The only treatment applied was PG administered as fed to the cows in SCK status as reported later.

### Milk Analysis

The milk samples were preheated at 40°C and analyzed using a Milkoscan 7 combined with a Fossomatic FC for SCC analysis (FOSS, Hilleroed, Denmark) in the laboratory of the Istituto Zooprofilattico Sperimentale della Sicilia, A. Mirri (Ragusa, Italia). The MilkoScan and the Fossomatic use a compact single-beam infrared system, based on FTIR (Fourier Transform Infrared Spectroscopy) technology (ISO 9622:2013-IDF 141:2013 Milk and Liquid milk products-Guidelines for the application of mid-infrared spectrometry) exploring the entire infrared spectrum (wavelength of 2 and 10 μm), thus, improving the accuracy in reading samples. The infrared evaluation of milk components allows to calculate the: fat (% g/100 ml), protein (% g/100 ml), lactose (% g/100 ml), casein (% g/100 ml), urea (mg/dl), somatic cell count (SCC) (num./ml), BHB (mmol/L), acetone (mmol/L). The threshold used to classify the positive sample of SCK, based on the individual milk BHB concentration, was ≥0.10 mmol/L. This parameter is reported in the FOSS Customer Support manual, 0102 6343/Rev.7 “Ketosis Prediction Models: Acetone and BHB” (18).

#### Propylene Glycol Treatment

The Propylene Glycol (PG) treatments and doses were decided according to the BHB values, with the collaboration of the farm veterinarians. The treatment was administered once a day together with the feed based on the weight of the animal. In each farm, the herd was divided into two groups (SCK positive cows, treated and SCK negative cows, untreated). A dose of 400 g/head as feed was administered to all the SCK-positive cows with the values of BHB > 0.10 mmol/L in milk, no SCK-positive cows were left untreated. The SCK-positive cows were treated once a day from the 2nd to 6th consecutive day when the sampled milk was used to evaluate the BHB values, if the BHB value was not back in the range of normal (<0.10 mmol/L) at the following milk sampling, the treatment was repeated again (19), and the cows were defined as uncured, otherwise, the cows defined as cured.

#### Statistical Analysis

The data on cows, milk analysis, and treatment were collected in a database. Data description, including prevalence estimation with confidence intervals, were performed on XLSTAT 2020.5.1 (Addinsoft, New York, USA). The analysis of frequency distributions (i.e., sampling class distribution, the efficacy of the treatments) was performed by χ^2^ test on XLSTAT 2020.5.1 (Addinsoft, New York, USA). An analysis for repeated measures (HPMIXED procedure; SAS Institute Inc., Cary, NC, USA) was used to assess the association of milk quality parameters with the breed, parity, DIM, and SCK as fixed effects. Herds and cows were included in the model as random effects.

The statistical model, for all the parameters, was the following:


$$\begin{array}{l} Y_{mopq}=\text{μ}+\;Herd_{m}\;+Cow_{n}+Breed_{o}+Parity_{p}+DIM_{q}\\ \;+\;SCK_{r}+SCK_{r}*Breed_{o}\\ \;+SCK_{r}*parity_{p}\;+e_{nopqr} \end{array}$$

where: Y_mnopq_ is the observed value for the different milk components (fat, protein, lactose, casein, and SCC); μ is the overall mean; Herd_m_ is the random effect of the *m*th herd (*m* = 1–22); Cow_n_ is the random effect of the *n*th animal (*p* = 1–1,588); Breed_o_ is the fixed effect of the *o*th class of breed (*o* = Holstein Fresian, Brown Swiss, mixed breeds); Parity_*p*_ is the fixed effect of the *p*th class of parity (*p* = 1–5); DIM_*q*_ is the fixed effect of the *q*th class of days in milk (*q* = A–E); SCK_*r*_ is the fixed effect of the *r*th class of SCK (*r* = 0, no; 1, yes); SCK_r_*Breed_*o*_ is the fixed effect of the interaction between the *r*th class of SCK and the *o*th class of Breed; SCK_r_*Parity_*p*_ is the fixed effect of the interaction between the *q*th class of SCK and the *p*th class of Parity; e_mnopqi_ is the residual error.

The analysis of the influence of treatment on milk quality and yield was assessed by a general linear model (GLM procedure; SAS Institute Inc., Cary, NC, USA) comparing the results of cured and uncured cows after one treatment cycle with the following model:


$$\begin{array}{l} Y_{nopqr}=\text{μ}+\;Sampling_{n}\;+Breed_{o}+Parity_{p}+TreatEff_{q}\\ \;+e_{nopq} \end{array}$$

where: Y_nopqr_ is the observed value for the different milk components (fat, protein, lactose, casein, and SCC); μ is the overall mean; Sampling_*n*_ is the fixed effect of sampling time (*n* = A to E); Breed_*o*_ is the fixed effect of the *o*th class of breed (*o* = Holstein Fresian, Brown Swiss, mixed breeds); Parity_*p*_ is the fixed effect of the *p*th class of parity (*p* = 1–5); TreatEff_*r*_ is the outcome of treatment application (*q* = cured, uncured); e_nopq_ is the residual error. Somatic cell count was log_10_ transformed (SCC) to obtain a normal distribution of this variable.

## Results

Samples were collected between day 1 and day 80 from calving during the lactation period (DIM), and they were classified into 5 classes as reported in Table 1, where are reported BHB-positive and BHB-negative results. The threshold was set at 0.10 mmol/L. The values higher or equal to the threshold were defined as BHB-positive of the subclinical or clinical ketosis as reported by de Roos et al. (18). Samples between 1st and 21st DIM (A–C classes) were collected to identify the presence of SCK or to check the efficacy of the treatment when applied. Samples from the 22nd DIM up to 80th (D and E classes) were mainly collected to check the efficacy of treatment of cows with SCK, thus, explaining the large decrease in the number of samples in these classes. The statistical analysis (χ^2^ test) on the frequency of BHB outcomes among the five sampling classes showed to be statistically significant (*p* < 0.0001). Comparison of frequencies within each class showed that class D did not show a significant difference in prevalence. Cows between 10 and 21 DIM have a higher than expected frequency of samples with BHB-negative values (χ^2^ test, *p* < 0.05); whereas cows in the first 9 DIM and with > 30 DIM showed a higher than expected frequency of BHB-positive outcome (χ^2^ test, *p* < 0.05).

**Table 1:** Description of sample frequencies by days in milk and relative BHB status.

**Class**	**Days**	**Samples**	**Frequency**	**Prevalence (%)**
	**in milk**	**no**.	**(C.I.) %**	**BHB positive**
				**(95% C.I., %)^1^**
A	1–9	1,091	27.4 (26.0–28.7)	41.5%^b^ (38.6–44.5)
B	10–15	1,054	26.4 (25.1–27.8)	20.1%^B^ (17.8–22.6)
C	16–21	1,017	25.5 (24.4–26.7)	17.1%^B^ (14.9–19.5)
D	22–30	464	11.6 (10.7–12.7)	29.3%^a^ (25.3–33.6)
E	>30	363	9.1 (8.2–10.0)	35.8%^b^ (31.0–40.8)

1 *Columns with a different letter are statistically different (α = 0.05). capital letters denote a frequency lower than expected in BHB-positive samples. lower letters denote a frequency higher than expected in BHB positive samples as estimate by χ^2^ statistics*.

### Factors Affecting Milk Quality and Yield

The statistical analysis showed that most of the factors considered had a significant effect on milk components and on SCC (Table 2). Only sampling and the interaction of BHB and breed or parity showed non-significant differences. The differences between mean values classified by the BHB results are reported in Table 3, showing as BHB-positive samples had significantly higher means for fat proportion, and SCC, while a significantly lower mean for protein proportion was observed. Table 4 reports the mean SCC values classified by BHB and for the five sampling classes, and in all the sampling periods a significantly higher SCC value was observed in BHB samples ≥0.10 mmol/L.

**Table 2:** Proportion of variance for random effects (herd, cow) and significance of fixed effects on milk components and SCC resulting from HPMIXED analysis for the repeated measurements.

**Response variable**	**Herd**	**Cow**	**Breed**	**Parity**	**Sampling**	**SK^a^, ^d^**	SK^*^	**SK**
							**breed**	^*^parity
Fat (%)	30.4%	4.1%	n.s.^c^	<0.0001	n.s.	0.0089	n.s.	n.s.
Protein (%)	4.0%	15.0%	<0.0001	<0.0001	n.s.	<0.0001	n.s.	n.s.
Lactose (%)	4.3%	16.6%	0.0008	<0.0001	<0.0001	<0.0001	0.0523	n.s.
Casein (%)	4.6%	13.0%	<0.0001	<0.0001	n.s.	<0.0001	n.s.	n.s.
SCC^d^ (log_10_)	4.81%	24.1%	<0.0482	<0.0001	<0.0001	<0.0001	n.s.	0.0123

a *SK = samples with BHB value ≥ 0.10 (mmol/L)*.

b *SK, subclinical ketosis;*

c *n.s., statistically not significant;*

d *SCC, somatic cell count*.

**Table 3:** Estimated means (± std.err.) for milk components values and somatic cell count (SCC) in relation to the BHB values (threshold = 0.10 mmol/L) resulting from HPMIXED analysis for repeated measurements (see Table 2).

**Subclinical**	**Fat (%) ±**	**Protein (%) ±**	**Lactose (%) ±**	**Casein (%) ±**	**SCC^b^ (log_**10**_/ml) ±**
**ketosis**	**std.err**.	**std.err**.	**std.err**.	**std.err**.	**std.err**.
BHB^a^<	3.73 ± 0.25	3.52 ± 0.03	4.82 ± 0.02	2.72 ± 0.02	4.83 ± 0.05
BHB ≥	4.03 ± 0.26	3.40 ± 0.03	4.72 ± 0.03	2.61 ± 0.03	5.11 ± 0.06

a *BHB, β-hydroxybutyrate;*

b *SCC, somatic cell count*.

**Table 4:** SCC (log_10_/ml) estimated means (± std.err.) classified by BHB values (threshold = 0.10 mmol/L) and parities resulting from HPMIXED analysis for repeated measurements (see Table 2).

**Subclinical ketosis**	**1st parity**	**2nd parity**	**3rd parity**	**4th parity**	**>4th parity**
BHB^a^<	4.86 ± 0.05^***^	4.72 ± 0.05^***^	4.85 ± 0.06^***^	4.87 ± 0.08^***^	4.88 ± 0.06^**^
BHB ≥	5.16 ± 0.06	4.95 ± 0.07	5.11 ± 0.07	5.31 ± 0.06	5.03 ± 0.08

*** *p < 0.0001*.

** *p < 0.05*.

a *BHB, β-hydroxybutyrate*.

#### Treatment Effect

Propylene glycol reduces the triacyglycerol (TG) content in the liver and the concentrations of ketone bodies in milk and hence, having anti-ketogenic properties (20). Overall, 1,100 cows (27.6%) were treated during the follow-up period of the study. The number of treated cows among breeds was statistically not different (Holstein Friesian: 27.6%; Brown Swiss: 26.6%; and Mixed breeds: 28.6%). Almost all the cows with BHB ≥ 0.10 mmol/L were treated between 1 and 21 DIM, while the treatment number significantly decreased afterward (Table 5). A significantly high proportion of treatment was observed in the first 9 DIM.

**Table 5:** Percentage of treated cows during the five sampling periods.

**Treatment**	**Frequency**	**Sampling period**					
		**A (1–9 DIM)**	**B (10–15 DIM)**	**C (16–21 DIM)**	**D (22–30 DIM)**	**E (>30 DIM)**	**Total**
No	Count	641^a^, ^1^	842^b^	843^b^	329^c^	234^c^	2,889
	% (95% CI^1^)	22.2 (20.7–23.7)	29.1 (27.5–30.8)	29.2 (27.5–30.9)	11.4 (10.3–12.6)	8.1 (7.1–9.1)	82.4 (71.0–73.8)
Yes	Count	450^a^	212^b^	174^b^	135^c^	129^c^	1.100
	% (95% CI)	40.9 (38.0–43.9)	19.3 (17.0–21.7)	15.8 (13.8–18.0)	12.3 (10.4–14.3)	11.7 (9.9–13.7)	27.6 (26.2–29.0)

1 *Different superscript letters along rows mean significant different values at α = 0.05*.

1 *CI, confidence interval*.

**Figure 1:**
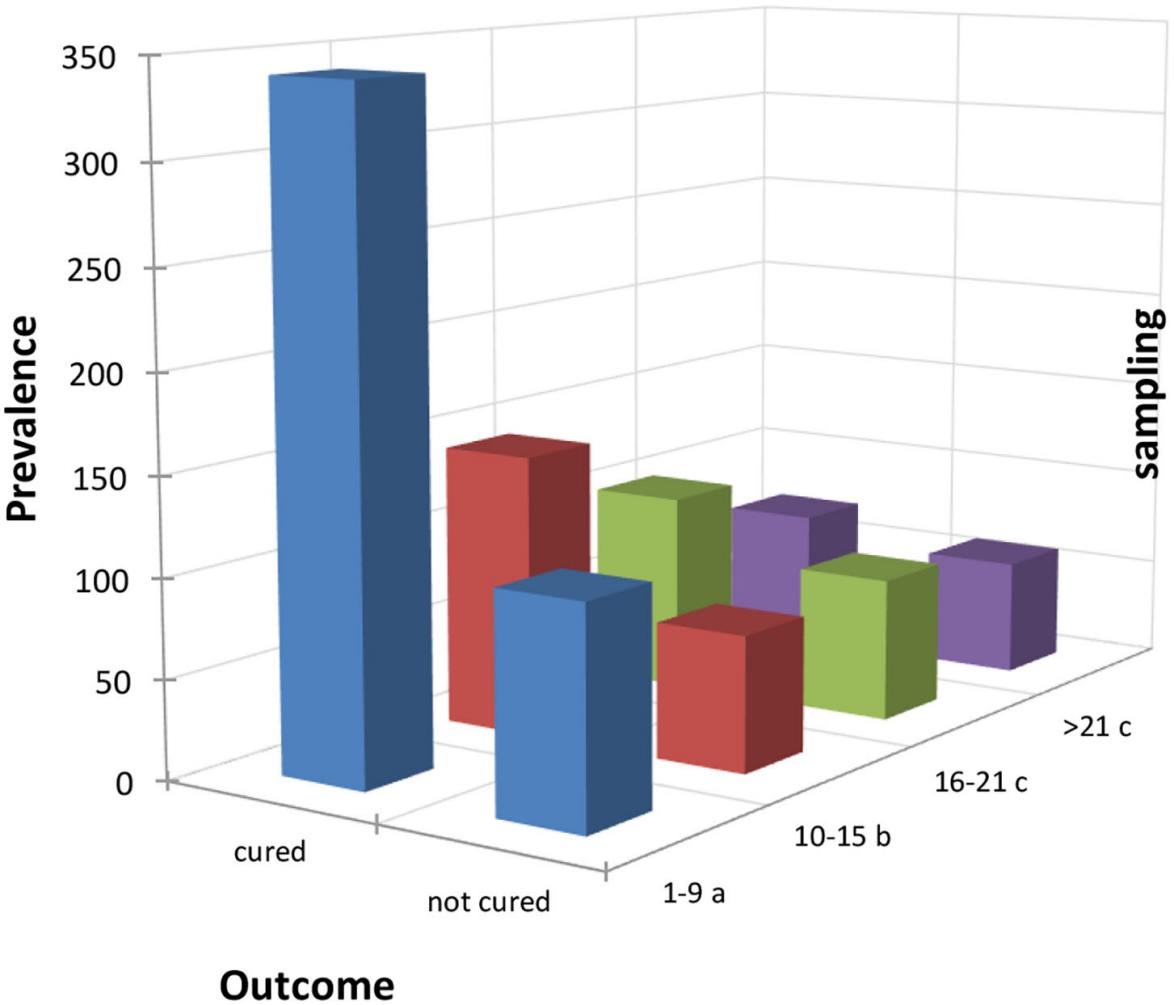
Distribution of the outcomes of the treated cows within the four sampling periods considered (periods 4 and 5 were added up to have a consistent number of samples). Letters denote the differences between expected and observed results based on χ^2^ test as follows: (a) significantly higher frequency of cure at α = 0.05; (b) no statistical differences, (c) significantly lower frequency of cure at α = 0.05.

Cows were considered cured when the BHB value dropped below the threshold value (i.e., BHB < 0.10 mmol/L) after treatment based on PG. The analysis of frequency of cured cows showed 67.85% of cows were cured with significant differences among DIM. Indeed, cows treated within 1–9 DIM showed a cure rate of 75.56%, treatment within 10–15 DIM had a cure rate of 67.45%, 58.05% within 16–21 DIM, and 54.26% within 22 and 30 DIM. Statistical analysis suggested that the prevalence of cured cows was significantly higher when they were treated in the early lactation period (1–9 DIM) and lower when they were treated after 21 DIM as shown in Figure 1.

The effects of treatment outcome and the factors considered in the study were analyzed by the GLM procedure. The results reported in Table 6 showed that the cured cows had a significant improvement on all the milk components except for the fat proportions. As reported in Table 7, the cured cows had significant higher proportion of protein, lactose, and casein compared to uncured cows; whereas SCC was significantly lower in the cured ones compared with the uncured cows.

**Table 6:** Significance of fixed effects on milk components and SCC resulting from ANOVA analysis.

**Response variable**	**Treatment outcome**	**Herd**	**Breed**	**Parity**	**Sampling**
Fat (%)	n.s.^a^	<0.0001	n.s.	n.s.	n.s.
Protein (%)	0.0255	0.0002	0.0098	<0.0001	<0.0001
Lactose (%)	0.0001	0.0058	n.s.	n.s.	0.0005
Casein (%)	0.0061	<0.0001	0.0191	<0.0001	<0.0001
SCC^b^ (log_10_)	<0.0001	<0.0001	n.s.	<0.0001	n.s.

a *n.s., statistically not significant;*

b *SCC, somatic cell count*.

**Table 7:** Estimated means (±std. err.) of milk components and SCC based on treatment outcome resulting from ANOVA analysis for repeated measurements (see Table 6).

**Treatment outcome**	**Fat%**	**Protein%**	**Lactose%**	**Casein%**	**SCC^b^ (log_**10**_/ml)**
Cured	3.47 ± 0.24	3.28 ± 0.04	4.93 ± 0.04	2.56 ± 0.03	4.75 ± 0.08
Not cured	3.72 ± 0.25	3.22 ± 0.04	4.82 ± 0.05	2.50 ± 0.03	5.03 ± 0.08
*P* (difference)	n.s.^a^	0.0255	0.0001	0.0061	<0.0001

a *n.s., statistically not significant;*

b *SCC, somatic cell count*.

## Discussion

The early detection of SCK could be an important factor to prevent the clinical ketosis in order to limit as much as possible the economic losses due to this disease. Often, SCK goes undiagnosed after calving, causing a reduction in milk yield and quality likewise to those caused by the clinical ketosis. Several studies report that the frequency of undiagnosed SCK varies between 15 and 40% of herd population (15, 17). This pathology is considered as a gateway for other metabolic and infectious disorders such as metritis, mastitis, clinical ketosis, hypocalcemia, and dislocation of the abomasum (11, 21, 22). Although some studies discourage the use of mid-infrared spectrometry analysis of individual milk sample as the only method for detecting hyperketonemia (23) milk remains the most promising matrix, because it allows a complete screening of the health of the animal and the herd being easy to sample and analyze (24). In addition, commercial farmers are used to collect the milk samples monthly, and this practice do not allow to perform a complete screening of the herd. However, individual milk sample analysis shows also the quality related to the fat proportion, the protein proportion, and the value of SCC, useful in this study for detecting hyperketonemia.

This cross-sectional study aimed to estimate the prevalence of undiagnosed SKC in 22 farms of the southeast of Sicily through quantification of BHB values in individual milk. Serum or plasma samples were not collected or analyzed because a comparison of BHB values among different biological samples was out of the scope of this study. Moreover, there were also evaluated the quantity of fat, protein, lactose, casein, urea, and somatic cell count (SCC) in BHB positive cows. The relationship between BHB concentrations and milk components suggests a correlation between hyperketonemia and an increase in the percentage of fat related to a decrease in the percentage of protein. In this study, high values of milk BHB were detected mostly in early lactation, indeed the BHB-positive samples have a statistically significant higher frequency than that expected in the case of sampling times A and E, while the frequency was lower than expected in sampling times B and C. The higher frequency in the first 9 days coincide with a peak of incidence of blood SCK, as reported by McArt et al. (16) and highlights the importance of post-partum period, in which it is recommended monitoring the values of BHB in milk to prevent clinical ketosis and design an individual therapy for cows in SCK. This study also allows to find a correlation in BHB-elevated samples of fat proportion and SSC average, both statistically higher than negative ones, in spite of the protein proportion significantly lower. Some studies have already reported associations of a higher percentage of fat and a lower percentage of protein in milk, with an increase in ketones concentrations (25, 26). Therefore, the variations in the fat–protein ratio, may be an early warning sign for forthcoming disorders, including SCK (27, 28). However, few studies have analyzed the relationship between BHB and SCC values. These studies show an increase of SCC in cows with high BHB values (29, 30) as found in this study. The increase of SCC values in milk may be addressed to the biochemical and inflammatory changes induced by ketone bodies. Indeed, the production of ketone bodies by the liver, at the beginning of lactation induces oxidative stress with consequent loss of cellular tissue (31). The pro-inflammatory cytokines, produced by the liver, increase the permeability of various tissues such as the breast parenchyma, making it susceptible to the potential penetration of pathogens. The presence inflammatory process together with the presence of pathogens, inevitably leads to an increase in SCC, in particular the neutrophils (32).

This study also aimed to demonstrate that treatment based on PG may balance the levels of BHB and reduce the negative effects on milk quality and yield. PG prevents fatty liver syndrome and accelerates the recovery of estrus cycles (33). Recent studies (19) have shown that supplemental PG to dairy cows during the perinatal period is an effective method for alleviating negative energy balance. The immune response and NEB are strictly correlated (34, 35), therefore, nutrition management during the post-partum period should be oriented toward NEB reduction (36). Furthermore, PG improves milk production, reproduction, and immune performance by improving plasma glucose and liver function in cows with SCK and reduces the quantity of fat in milk. In this study, 67.85% of cows showed values lower than 0.10 mmol/L after PG treatment, with significant differences in the number of recovered cows among the time of treatment. Statistical analysis showed that the frequency of recovered cows was higher than expected when the treatment was administered within the first 9 days of post-partum and lower than expected when applied after 22 days. The treatment had a significant effect on all components of the milk except the proportions of fat, while it significantly influenced the proportion of protein, lactose, and casein, significantly higher in the treated cows; SCC was significantly lower in treated than in the untreated cows.

These results contribute to raising awareness to SCK and aim to encourage the use of individual milk samples to make an early diagnosis of SCK. The sampling method is easy to do and cheap; the milk analysis (Fourier Transform Infrared Spectroscopy) is also low cost, but simultaneously, very accurate. The study suggests that the early diagnosis of SCK could be sustainable because the cost of diagnosis through milk analysis may be covered by the loss income caused by the reduction of the milk yield due to SCK (7, 37).

## Conclusions

Subclinical ketosis is often misdiagnosed until the onset of clinical one, despite its high prevalence in many dairy herds. The economic losses related to this disease are very serious compared with the low-cost analysis to diagnosis it. Individual milk analysis thought Fourier Transform Infrared Spectroscopy represents the cheapest way to monitor the levels of β-hydroxybutyrate while the early treatment with propylene glycol could rebalance the levels of β-hydroxybutyrate and positively influence the other milk components. This cross-sectional study allowed to assess, for the first time, the prevalence of SCK on Sicilian dairy herds and to confirm that an early diagnosis of subclinical ketosis followed by a propylene glycol treatment might decrease the effects of this disease on milk total production improving the milk yield and quality (protein, lactose, casein, and somatic cell).

## Data Availability Statement

The original contributions presented in the study are included in the article/supplementary material, further inquiries can be directed to the corresponding author.

## Ethics Statement

Ethical review and approval was not required for the animal study because samples of milk were collected by researcher in the farms and analyzed at Istituto Zooprofilattico Sperimentale della Sicilia for diagnostic purposes. Written informed consent was obtained from the owners for the participation of their animals in this study.

## Author Contributions

The conceptualization of this study was done by GC, FA, and FL. The investigation was in charge of SA, MD, and RS. The data curation and statistical analysis was developed by AZ. AS and FL wrote the original draft. All co-authors contributed to the management of this case, interpretation of data, and carefully revised the manuscript. All authors have read and agreed to the published version of the manuscript.

## Funding

This open access publication fee was supported by fund of the Istituto Zooprofilattico Sperimentale della Sicilia.

## Conflict of Interest

SA and MD were employed by company Leocata Mangimi. The remaining authors declare that the research was conducted in the absence of any commercial or financial relationships that could be construed as a potential conflict of interest.

## Publisher's Note

All claims expressed in this article are solely those of the authors and do not necessarily represent those of their affiliated organizations, or those of the publisher, the editors and the reviewers. Any product that may be evaluated in this article, or claim that may be made by its manufacturer, is not guaranteed or endorsed by the publisher.
